# Long Leukocyte Telomere Length Is Associated with Increased Risks of Soft Tissue Sarcoma: A Mendelian Randomization Study

**DOI:** 10.3390/cancers12030594

**Published:** 2020-03-05

**Authors:** Yifan Xu, Junfeng Xu, Haidee Chancoco, Maosheng Huang, Keila E. Torres, Jian Gu

**Affiliations:** 1 Departments of Epidemiology, The University of Texas MD Anderson Cancer Center, Houston, TX 77030, USA; 2 Departments of Surgical Oncology, The University of Texas MD Anderson Cancer Center, Houston, TX 77030, USA

**Keywords:** soft tissue sarcoma, cancer risk, leukocyte telomere length, Mendelian randomization

## Abstract

Background: Leukocyte telomere length (LTL) has been associated with the risks of several cancers in observational studies. Mendelian randomization (MR) studies, using genetic variants as instrumental variables, have also shown associations of genetically predicted LTL with cancer risks. In this study, we performed the first MR analysis on soft tissue sarcoma (STS) to investigate the causal relationship between LTL and the risk of STS. Methods: Genotypes from eleven LTL-associated single nucleotide polymorphisms (SNPs) in 821 STS cases and 851 cancer-free controls were aggregated into a weighted genetic risk score (GRS) to predict LTL. Multivariate logistic regression was used to assess the association of STS risk with individual SNPs and aggregated GRS. Results: Four SNPs displayed evidence for an individual association between long LTL-conferring allele and increased STS risk: rs7675998 (odds ratio (OR) = 1.21, 95% confidence interval (CI) = 1.02–1.43), rs9420907 (OR = 1.31, 95% CI = 1.08–1.59), rs8105767 (OR = 1.18, 95% CI = 1.02–1.37), and rs412658 (OR = 1.18, 95% CI = 1.02–1.36). Moreover, longer genetically predicted LTL, calculated as GRS, was strongly associated with an increased risk of STS (OR = 1.44, 95% CI = 1.18–1.75, *p* < 0.001), and there was a significant dose-response association (*p* for trend <0.001 in tertile and quartile analyses). The association of longer LTL with higher STS risk was more evident in women than in men. In stratified analyses by major STS subtypes, longer LTL was significantly associated with higher risks of leiomyosarcoma and gastrointestinal stromal tumors. Conclusions: Longer LTL is associated with increased risks of STS.

## 1. Introduction

Soft tissue sarcomas (STS) constitute a heterogeneous combination of malignancy derived from mesenchymal tissue [1,2,3]. Overall, STS account for 1% of adult malignancies and 15% of pediatric malignancies [4]. STS can arise from any part of the body and are most commonly derived from extremities [1]. The overall 5-year survival rate is about 65%, and the 5-year survival rates for localized, regional, and distant STS were 81.2%, 57.4%, and 15.9%, respectively [5]. Most STS are sporadic, and their etiology and genetic susceptibility are not well understood. STS are an aging-related disease with a median age at diagnosis of 60 years and the median age of death being 66 years [6]. Radiation exposure is a strong risk factor for STS, and viral infection (e.g., HPV and HIV) predisposes to certain subtypes of STS [2,3]. Other potential risk factors such as occupational exposures to herbicides and chlorophenols need more compelling evidence [2,3]. A few inherited genetic syndromes, such as neurofibromatosis type 1 (NF1), Li–Fraumeni syndrome (LFS), and retinoblastoma (Rb), have been associated with increased risks of STS [2,3]. No common genetic variant has been unequivocally linked to adult STS susceptibility due to the rarity and heterogeneity of this disease [7].

Telomeres are hexameric nucleotide repeats and protein complex capping both ends of eukaryotic chromosome arms [8,9]. Telomeres prevent the termini of linear chromosomes from fusion and degradation [10,11]. The shortening of telomeres is recognized as a ‘molecular clock’ that curbs organisms’ age, which will finally cause chromosomal instability, cellular senescence, cell cycle arrest, and eventually apoptosis [11,12]. Telomerase is activated in 90% of tumors and contributes to tumor cells’ immortal growth properties in the presence of shortened telomeres [13].

Telomere length is often measured in readily accessible leukocyte DNA, and leukocyte telomere length (LTL) is highly correlated with the telomere length in other tissues [14]. LTL is under strong genetic control, with an estimated heritability of up to 80% from classic twin studies [15,16]. Although LTL is generally inversely correlated with age, there is a considerable interindividual variation of LTL among people of the same ages [17,18]. In addition to genetic factors, LTL can also be shortened by environmental factors such as smoking and occupational exposure to harmful chemicals [19,20,21]. The interindividual variation of LTL has been shown to contribute to genetic susceptibility to cancer and other diseases [22,23,24,25,26,27]. Earlier retrospective case-control studies suggested that short LTL was a risk factor for some cancers, but later large prospective studies and recent Mendelian randomization studies using genetically predicted LTL have increasingly found that long LTL was a risk factor for a number of cancers, such as melanoma, B-cell lymphoma, lung adenocarcinoma, glioma, renal cell carcinoma, and osteosarcoma [24,25,26,27,28,29,30,31,32,33,34,35,36,37].

We previously reported that longer LTL, measured by the standard real-time quantitative PCR (qPCR) method, was associated with a higher risk of STS in a pilot case-control study of 137 pairs of STS cases and controls [38]. However, the assessment of disease association of an intermediate phenotypic biomarker such as LTL in a retrospective case-control study is subjected to several limitations, including reverse causation, environmental confounding, treatment confounding, variability in sample preparation, and variability in technical measurement. Mendelian randomization (MR) is an approach using common genetic variations as instruments to study the causal relations between risk factor/intermediate biomarkers and health outcomes in observational data, which is less affected by confounding, reverse causation, and technical variability [39]. There are three assumptions in MR studies: (1) the selected genetic variants are associated with the studied risk factor/biomarker; (2) the genetic variants are independent of other confounding factors that are associated with the selected risk factor/biomarker and disease; (3) the genetic variants only influence disease risk through their effects on the risk factor/biomarker. Eleven independent single nucleotide polymorphisms (SNPs) have been unequivocally identified to be associated with LTL by large scale genome-wide association studies (GWAS) [40,41,42]. Numerous MR studies have used these SNPs to assess genetically predicted LTL and risks of diseases, including cancers [27,28,29,30,31,32,33,34,35,36,37,39]. However, no MR study of LTL and STS risk has been reported to date. In this study, we used a large case-control study and applied an MR approach to test the hypothesis that genetically predicted longer LTL is associated with increased risks of STS. The large sample size also allowed us to perform stratified analyses of the major histological subtypes of STS.

## 2. Results

### 2.1. Characteristics of the Study Population

The distribution of selected characteristics of the 821 STS patients and 851 age- and gender-matched controls are shown in Table 1. All the participants were Caucasians. The average diagnosis age for STS patients was 56.39 and for controls was 57. There are slightly more females than males in both cases and controls. The major histological subtypes were leiomyosarcoma (33.1%), gastrointestinal stromal tumors (GIST, 26.8%), liposarcoma (22.0%), and angiosarcoma (7.3%).

### 2.2. Association between Individual SNP and STS Risk

The association between each individual LTL-associated SNP and STS risk is shown in Table 2. Among the 11 SNPs, four were nominally significantly associated with STS risk: *NAF1* rs7675998 (OR = 1.21, 95% CI = 1.02–1.43, *p* = 0.026), *OBFC1* rs9420907 (OR = 1.31, 95% CI = 1.08–1.59, *p* = 0.007), *ZNF208* rs8105767 (OR = 1.18, 95% CI = 1.02–1.37, *p* = 0.030), and *ZNF676* rs412658 (OR = 1.18, 95% CI = 1.02–1.36, *p* = 0.025). In all these 4 SNPs, alleles related to longer LTL were associated with increased risks of STS.

### 2.3. Association between GRS and STS Risk

We then constructed a GRS using the 11 SNPs and tested the association of this GRS with STS risk (Table 3). Dichotomized at the median GRS in controls, individuals with higher GRS (longer LTL) were associated with a 1.44-fold increased risk of STS (95% CI = 1.18–1.75, *p* = 3.63 × 10^−4^). In the tertile analysis, the ORs for the risk of STS in the medium and longest tertile groups were 1.35 (95% CI = 1.05–1.73) and 1.56 (95% CI = 1.22–1.99, respectively (*p* for trend 4.36 × 10^−4^). In quartile analysis, the ORs for the risk of STS were gradually elevated with the rising of GRS and the ORs in 2nd, 3rd, and 4th (longest) quartile groups were 1.25 (95% CI = 0.93–1.68), 1.43 (95% CI = 1.07–1.92), and 1.79 (95% CI = 1.35–2.38), respectively (*p* for trend 3.20 × 10^−5^).

### 2.4. Gender-Specific Association of LTL GRS with STS

We then performed stratified analyses to determine whether the association of LTL GRS with the risk of STS was different in men and women (Table 4). The association was highly significant in women (OR = 1.68, 95% CI = 1.26–2.23, *p* = 3.54 × 10^−4^), but not in men (OR = 1.14, 95% CI = 0.85–1.53, *p* = 0.39).

### 2.5. Association of LTL GRS with Histologic Subtypes

We next performed stratified analyses to analyze the associations of LTL GRS with the risks of major STS subtypes (Table 5). The most significant association was observed in GIST: longer LTL conferred a 2.2-fold increased risk (95% CI = 1.58–3.06, *p* = 3.03 × 10^-6^). The association was also significant in leiomyosarcoma (OR = 1.36, 95% CI = 1.02–1.83, *p* = 0.038), but not in liposarcoma (OR = 1.05, 95% CI = 0.75–1.47, *p* = 0.772) or angiosarcoma (OR = 1.19, 95% CI = 0.70–2.02).

## 3. Discussion

In this case-control study, we used a two-sample MR approach to assess the associations between genetically predicted LTL and the risk of STS. We found a strong association between higher GRS (longer LTL) and an increased risk of STS with a dose–response relationship. Stratified analyses found the significant associations were more evident in GIST and leiomyosarcoma. This is the first MR study to show that long LTL predisposes to the development of STS.

There have been numerous epidemiologic investigations that assessed the association of LTL with the risk of different cancers. Earlier small, retrospective case-control studies produced inconsistent results, although most retrospective studies showed that short LTL predisposes to cancer development due to reverse causation [43,44,45,46,47,48,49,50]. Later large prospective studies have provided evidence for cancer-type-specific associations; both short and long LTL can predispose to cancer development [24,26,51,52]. Recent MR studies have further shown that genetically predicted long LTL is associated with increased risks of several cancers, including B-cell lymphoma, melanoma, lung adenocarcinoma, neuroblastoma, glioma, meningioma, and osteosarcoma [27,28,29,30,31,32,33]. Together with the results of the previous case-control study that long LTL as measured by qPCR conferred an increased risk of STS [38], our data provide compelling evidence for the association of long LTL with increased risks of STS.

The biological mechanisms of the association between longer LTL and higher cancer risks are not well understood. Recent studies have suggested that telomere dysfunction may have a binary effect on carcinogenesis [13]: both short and long telomeres can facilitate cancer development. Extremely shortened telomere length could result in increased chromosome end-to-end joining, thereby cause genome instability and malignant transformation. On the other hand, very long telomeres could increase cancer risk by allowing continued cellular proliferation and delaying cellular senescence and apoptosis, hence providing an environment that factors the accumulation of genetic lesions. Another potential biological explanation for the strong association between long LTL and high STS risk may be related to the distinct tissue origination of sarcomas. Unlike carcinomas, which originate from epithelial cells, sarcomas are malignant tumors derived from mesenchyme. Adult human tumors are predominantly epithelial carcinomas, whereas pediatric human tumors and murine tumors are primarily sarcomas and lymphomas. Longer telomere length in the pediatric population and mice than in adult human beings may partially explain this differential distribution of carcinoma and sarcoma [53]. Consistently, long LTL have been associated with increased risks of lymphoma, osteosarcoma, and STS [30,33,38,54]. Furthermore, Yan et al. previously reported long telomeres in tumor tissues of liposarcoma, leiomyosarcoma, and high-grade STS [55], in contrast to the generally shorter telomeres in epithelial tumors compared to adjacent normal tissues. 

In this study, we found that the association between long LTL and increased STS risk is highly significant in women but not in men. Interestingly, long LTL has been consistently associated with increased risks of most female cancers, including breast cancer [24,56,57], ovarian cancer [24,27], and endometrial cancer [24,27,58], suggesting that the estrogen regulation of telomeres may be linked to these female-specific associations. Estrogen can activate telomerase through direct binding to the promoter region of hTERT and prevent telomere shortening [59,60,61]. There was a positive correlation of the circulating estradiol with LTL in women [62], and LTL was longer in women who had a history of long-term hormone replacement therapy (HRT) than those without HRT [63]. The positive association between estrogen exposure and LTL may partially explain the association of longer LTL with increased risks of female STS.

The major strength of this study is the large sample size of histologically confirmed STS cases. Published prospective studies and MR studies of LTL and cancer risks have not included STS due to the rarity of the disease. We selected 11 SNPs that have been unequivocally associated with LTL through large scale GWAS. These SNPs are believed to meet the assumptions of MR and have been widely used in MR studies of LTL and disease risks. There are also a couple of limitations. First, the current GRS only explains approximately 2% of LTL variations. More powerful genetic instruments will increase statistical power and minimize confounding issues. Additional SNPs associated with LTL need to be identified to produce stronger GRS. Second, we could only perform stratified analyses on the major subtypes. STS represents a heterogeneous group containing more than 50 different histologic subtypes. Future large studies are needed to assess other STS histologic subtypes.

## 4. Materials and Methods

### 4.1. Study Population and Data Collection

STS patients were newly registered, histologically confirmed patients recruited from The University of Texas MD Anderson Cancer Center. The demographic and basic epidemiological information, including smoking, alcohol drinking, occupation, family history, and medical history, were obtained from the patient history database that all new patients filled when they registered into MD Anderson Cancer Center. Controls were healthy individuals with no cancer history who came to Kelsey-Seybold, one of the largest multispecialty physician groups in the Houston metropolitan area, for annual health checkups. Controls were frequency-matched to the cases by age, sex, and ethnicity. Demographic and epidemiological information of controls were collected by in-person interviews using standardized questionnaires. This study was approved by the institutional review board of MD Anderson Cancer Center on 7 April 2003 (ethic code: Lab03-0320), and all patients signed an informed consent form.

### 4.2. Genotyping

Genomic DNA was isolated from peripheral blood using the QIAamp blood DNA extraction kit (Qiagen, Valencia, CA, USA). All the genotyping was done in the Genotyping Core of MD Anderson Cancer Center using Illumina’s Infinium OncoArray-500K Beadchip. Genome Studio software (Illumina, San Diego, CA, USA) was utilized to analyze the genotyping data. The mean concordance rate of 2% replicated samples was 99.2%. We removed nonconcordant SNPs for analyses. All the samples had an overall SNP call rate >95%. Individual SNPs with minor allele frequency (MAF) <1% and call rate <90% were excluded for analysis. Imputation was performed using the Michigan Imputation Server (https://imputationserver.sph.umich.edu/), an online server that generates phased and imputed genotypes using the Haplotype Reference Consortium (HRC Version r1.1) reference panels [64]. Eleven independent SNPs were associated with LTL by large scale GWAS [40,41,42] and were used to construct a genetic risk score (GRS). Among these SNPs, four SNPs (rs10936599, rs2736100, rs9420907, and rs755017) were directly genotyped on OncoArray-500K, and the other seven were imputed with a high imputation accuracy (mean R^2^) of 0.96.

### 4.3. Mendelian Randomization (MR) Analysis and GRS Construction

A two-sample MR design was used to assess the association between genetically predicted LTL and the risk of STS. The SNP–LTL effects (estimate for each SNP) were derived from published GWAS [40,41,42]. A GRS was calculated using 11 LTL-associated SNPs according to the following formula:

$$GRSi=\overset{11}{\underset{j=1}{\sum }}WjXij$$

where GRS_i_ is the risk score for individual *i*. *x_ij_* (*x_ij_* = 0, 1, or 2) is the number of telomere length increasing alleles for the *j*-th SNP, and *w_j_* is the weight or effect coefficient (β estimate) for each SNP. A higher GRS value for an individual represents longer genetically inferred LTL. Weighted GRS counted the number of alleles associated with longer LTL that an individual carried across all 11 LTL-associated SNPs, with the addition of *w_j_* for each SNP. Weighted GRS produces higher specificity than unweighted GRS by assigning more weight to SNPs with stronger effects.

### 4.4. Statistical Analysis

We used χ^2^ test or Fisher’s exact test to compare allele frequencies of each individual SNP between cases and controls. We then analyzed the association between each SNP and the risk of STS using a multivariate logistic regression model adjusting for age and gender. To analyze the association between GRS and the risk of STS, we dichotomized GRS at the median value or categorized into three and four groups based on the tertile and quartile distribution in controls and used a multivariate logistic regression model to calculate odds ratio (OR) and corresponding 95% confidence interval (95% CI). We also collected smoking, BMI, and medical history data from both cases and controls. Adjusting for smoking, BMI, and chronic diseases such as diabetes and hypertension in multivariate logistic regression did not attenuate the risk estimate. Since these variables are not risk factors for STS, we did not include them in our final multivariate model. All data were analyzed using R software (v3.4.1) or STATA (v13, STATA Corp., College Station, TX, USA). The glm() function in R Package was used for unconditional logistic regression analysis. All *p* values were two-sided with *p* < 0.05 considered statistically significant.

## 5. Conclusions

This is the first MR study to evaluate the association of LTL and the risk of STS. Our data demonstrated that genetically predicted long LTL is strongly associated with an increased risk of STS, and the association was more evident in women than in men.

## Figures and Tables

**Table 1:** Selected characteristics of the study population.

Characteristics	Cases *n* = 821	Controls *n* = 851	*p* Value
**Age, years (mean, SD)**	56.39 (11.58)	57.00 (8.62)	0.22
**Gender, *n* (%)**			
Male	388 (47.26)	406 (47.71)	
Female	433 (52.74)	445 (52.29)	0.85
**Histology, *n* (%)**			
Leiomyosarcoma	272 (33.1)		
GIST *	220 (26.8)		
Liposarcoma	181 (22.0)		
Angiosarcoma	60 (7.3)		
Other	88 (10.7)		

* GIST: gastrointestinal stromal tumors.

**Table 2:** Individual leukocyte telomere length (LTL)-associated single nucleotide polymorphisms (SNPs) and soft tissue sarcoma (STS) risk.

SNP ID	Chr.	Position	Gene	Allele *	β *	EAF Case	EAF Control	OR ** (95% CI)	*p* Value
rs11125529	2	54475866	ACYP2	A/C	0.065	0.131	0.129	1.01 (0.82–1.24)	0.922
rs6772228	3	58376019	PXK	T/A	0.041	0.942	0.948	0.9 (0.67–1.2)	0.472
rs10936599	3	169492101	TERC	C/T	0.1	0.756	0.737	1.11 (0.94–1.3)	0.216
rs7675998	4	164007820	NAF1	G/A	0.048	0.8	0.766	1.21 (1.02–1.43)	0.026
rs2736100	5	1286516	TERT	C/A	0.085	0.487	0.48	1.02 (0.89–1.18)	0.730
rs9420907	10	105676465	OBFC1	C/A	0.142	0.164	0.13	1.31 (1.08–1.59)	0.007
rs3027234	17	8136092	CTC1	C/T	0.103	0.784	0.762	1.14 (0.96–1.34)	0.127
rs8105767	19	22215441	ZNF208	G/A	0.064	0.317	0.283	1.18 (1.02–1.37)	0.030
rs412658	19	22359440	ZNF676	T/C	0.086	0.382	0.344	1.18 (1.02–1.36)	0.025
rs6028466	20	38129002	DHX35	A/G	0.058	0.066	0.059	1.13 (0.85–1.5)	0.393
rs755017	20	62421622	ZBTB46	G/A	0.019	0.134	0.132	1.02 (0.84–1.25)	0.832

* Alleles are short allele/long allele. Short alleles are used as the reference allele and long allele as effect allele. EAF: effect allele frequency; estimates of SNP–LTL association were from published genome-wide association studies (GWAS). ** Adjusted by age and gender.

**Table 3:** Association of LTL genetic risk score (GRS) with STS risk.

LTL GRS	Control *n* (%)	Case *n* (%)	OR * (95% CI)	*p* Value	*p* for Trend
**Dichotomize**					
Short	410 (56.47)	316 (43.53)	1 (reference)		
Long	403 (47.30)	449 (52.70)	1.44 (1.18–1.75)	3.63 × 10^−4^	
**Tertile**					
Shortest	280 (58.09)	202 (41.91)	1 (reference)		
Medium	262 (50.58)	256 (49.42)	1.35 (1.05–1.73)	0.019	
Longest	271 (46.89)	307 (53.11)	1.56 (1.22–1.99)	3.85 × 10^−4^	4.36 × 10^−4^
**Quartile**					
1 (shortest)	207 (59.31)	142 (40.69)	1 (reference)		
2	203 (53.85)	174 (46.15)	1.25 (0.93–1.68)	0.139	
3	200 (50.38)	197 (49.62)	1.43 (1.07–1.92)	0.015	
4 (longest)	203 (44.62)	252 (55.38)	1.79 (1.35–2.38)	5.25 × 10^−5^	3.20 × 10^−5^

* Adjusted by age and gender.

**Table 4:** Gender-specific association of LTL GRS with the risk of STS.

LTL GRS	Control *n* (%)	Case *n* (%)	OR * (95% CI)	*p* Value
**Male**				
Short	175 (52.87)	156 (47.13)	1 (reference)	
Long	209 (49.76)	211 (50.24)	1.14 (0.85–1.53)	0.39
**Female**				
Short	235 (59.49)	160 (40.51)	1 (reference)	
Long	194 (44.91)	238 (55.09)	1.68 (1.26–2.23)	3.54 × 10^−4^

* Adjusted by age.

**Table 5:** Association of LTL genetic risk score (GRS) with the risk of major STS subtypes.

LTL GRS	Control *n* (%)	Case *n* (%)	OR * (95% CI)	*p* Value
**Leiomyosarcoma**				
Short	410 (78.85)	110 (21.15)	1 (reference)	
Long	403 (74.22)	140 (25.78)	1.36 (1.02–1.83)	0.038
**GIST**				
Short	410 (86.68)	63 (13.32)	1 (reference)	
Long	403 (74.63)	137 (25.37)	2.20 (1.58–3.06)	3.03 × 10^−6^
**Liposarcoma**				
Short	410 (83.33)	82 (16.67)	1 (reference)	
Long	403 (81.91)	89 (18.09)	1.05 (0.75–1.47)	0.772
**Angiosarcoma**				
Short	410 (93.82)	27 (6.18)	1 (reference)	
Long	403 (92.64)	32 (7.36)	1.19 (0.7–2.02)	0.528

* Adjusted by age and gender.
